# Cross-cultural translation and psychometric properties of the Persian version Manchester respiratory activities of daily living questionnaire (MRADLQ-P)

**DOI:** 10.1186/s12890-022-01920-4

**Published:** 2022-04-12

**Authors:** Kumars Eisapareh, Mahin Nazari, Hamidreza Mokarami

**Affiliations:** 1 Student Research Committee, Department of Health Promotion and Education, School of Health, Shiraz University of Medical Sciences, Shiraz, Iran; 2 Department of Health Promotion and Education, School of Health, Shiraz University of Medical Sciences, Shiraz, Iran; 3 Department of Ergonomics, School of Health Shiraz, University of Medical Sciences, Shiraz, Iran

**Keywords:** Cross-cultural, Psychometrics, MRADL, Translations, Persia

## Abstract

**Purpose:**

The present study was an attempt to investigate cross-cultural adaptability and evaluate the psychometric properties of the Persian version of the Manchester respiratory activities of daily living questionnaire ((MRADLQ-P).

**Patients and methods:**

In a cross-sectional study, we selected 260 patients with severe respiratory diseases who needed to be admitted to the respiratory wards of this city hospital. The process of cultural localization of the questionnaire was performed based on a standard and valid process. Psychometric properties of the instrument were confirmed based on face and content validity assessments, convergent validity, discriminative validity and internal consistency. Data collected by demographic questionnaire, MRADL questionnaire and work ability index. Data were analyzed by SPSS 22 using descriptive statistics (mean and standard deviation), Spearman correlation coefficient, Cronbach's alpha coefficient, and Mann–Whitney test.

**Results:**

The mean age of participants was 48.8 ± 20.1 years. 176 (71.5%) were male. face content validity including content validity index (CVI) was 0.82 and content validity ratio and it was good. The questionnaire was measured at the same time as the work ability index, which Mann–Whitney test showed that the questionnaire has good differential power. Cronbach's alpha coefficient of 0.9 indicates a very good reliability of the questionnaire.

**Conclusion:**

The results show that intercultural psychometrics of MRADL questionnaire has good validity, reliability, and differential power that can be a good tool for use in future studies. Also, the translation of this checklist included translation into the target language, backward translation of the Persian versions into the original language, and comparisons and ambiguities to obtain a final and acceptable version.

## Introduction

Respiratory diseases are one of the leading causes of death in the world [1], and one of this diseases is Chronic obstructive pulmonary disease(COPD) [2]. COPD is a chronic lung disorder characterized by irreversible and progressive flow restriction that causes physiological and functional impairment in patients, and with persistent reduction of airflow, it causes respiratory problems during exercise and during rest [3]. COPD has a significant impact on quality of life patients with respiratory problems [4]. These patients frequently report shortness of breath in doing daily activities [5, 6]. Daily life activities include the activities and tasks that people perform daily, and include taking care at home, outside the home, or both [2, 7]. Despite the many restrictions that COPD patients experience in their daily activities, the severity, type and level of daily activities in these patients are not known and there is no clear information of the factors affecting it. Also, different methods are used to measure the daily activities of life in patients with COPD, which are often not standard [8]. Using the right scale or instrument to measure daily activities of life contributes to determine the treatment and care program and related costs in a principled and reasonable manner, since each patient has different degrees of capability and restriction in performing daily activities [2, 9]. Questionnaires are convenient and accessible instruments that are easily available to patients and easily collect relevant data [2, 10]. The Manchester Respiratory Activities of Daily Living Questionnaire (MRADL) was developed by Yohannes et al. [11], and includes four areas of mobility (7 items), activities in the kitchen (4 items), domestic tasks (6 items), and leisure activities (4 items) [11]. MRADL is authentic, reliable, repeatable, and convenient questionnaire to complete it fast (10 min), Also, it can distinguish between COPD patients and healthy people and has good sensitivity [11, 12]. The Cronbach's alpha coefficient of the original version of this questionnaire is 0.91, indicating its very good internal consistency [11]. This questionnaire has already been translated into Portuguese for use in Brazil [13]. However, since MRADL is an instrument originally written in English, it should be translated into the target language and adapted to the social and cultural conditions of the target country [14, 15]. Our investigations suggest that the MRADL questionnaire has not been translated into Persian yet. In this regard, the present study was an attempt to investigate cross-cultural adaptability and evaluate the psychometric properties of the Persian version of this questionnaire.

## Material and methods

This cross-sectional study was conducted in Dezful, Iran. Participants included 260 patients with severe respiratory diseases who needed to be admitted to the respiratory wards of this city hospital. Inclusion criteria of the study included a patient with respiratory disease and need for hospitalization based on physician’s diagnosis, having the ability read a Persian questionnaire and complete the questionnaire on a self-report basis. After deleting the questionnaires with outlier and distorted data, the data of 246 participants were analyzed. To evaluate the psychometric properties of the questionnaire, it has been recommended to select 10 samples for each item [16, 17], so this number of samples was suitable for evaluating the psychometric properties of MRAD. The study was approved by the ethics committee under the code of IR.SUMS.REC.1398.972 at Shiraz University of Medical Sciences. Before distributing the questionnaires, a complete explanation was provided on the aim of study for the participants and all of them completed a written informed consent form to participate in the study.

### MRADL questionnaire

This questionnaire was designed and developed by Yohannes et al. [11], which includes four areas of mobility (7 items), activities in the kitchen (4 items), domestic tasks (6 items), and leisure activities (4 items); and scoring system is from 0 to 21, in which maximum score indicates physical incapability, also The answers in this questionnaire on the 4-point Likert scale include "not at all" to "with the help of others", "alone but with difficulty" and "alone and easily". Scoring is such that "not at all" and "with the help of others" are given a score of zero, and "alone but with difficulty" and "alone and easily" are given a score of one [11]. MRADL can distinguish between COPD patients and healthy people and has good sensitivity [11, 12].

#### The process of questionnaire translation

**Fig. 1:**
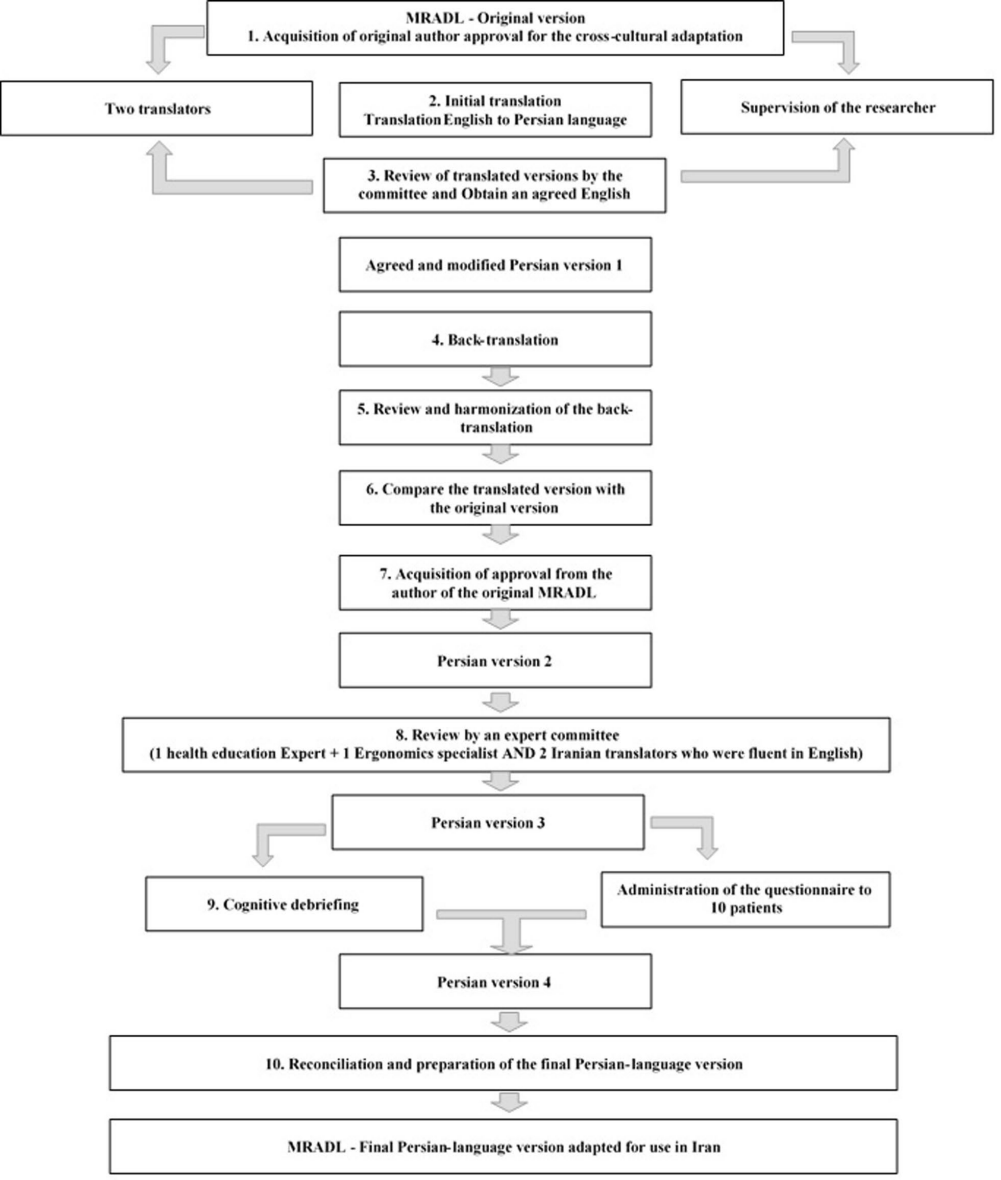
Cross-cultural translation and psychometric process diagram of MRDRL questionnaire in Persian

After obtaining permission for cross-cultural adaptability and compliance with MRADL usage rights, the cross-cultural adaptability process of the questionnaire was performed based on the guidelines accepted by Beaton et al. [18]. In the first step (Forward translation), the MRADL questionnaire was independently translated by two experts fluent in Persian-English. Then, translators and the research team compared the translated versions and discussed on ambiguous and unfamiliar terms, and the questionnaire items were examined and reviewed semantically, cross-cultural equivalence, and terms. In this step, a simple and appropriate version of the questionnaire was prepared while preserving semantic value of the original questionnaire. At the end of this step, a single temporary Persian version of the questionnaire was obtained. For example, the translation of the sentence “Do you bend over to stand?” into Persian (  ) was incomprehensible and changes were made to make it understandable, which in the backward translation conveys the meaning of the original sentence to the audience (Do you bend while standing (for better breathing)?); Or in the sentence “Do you wash your small clothes? (Underwear, socks, etc.)” we changed the small clothes to the “underwear clothes”. Also, the sentence “You take a bath yourself” was not understandable in Persian translation, so we changed it to a sentence of “You bathe alone”. Then, two other bilingual translators (who were not aware of the contents of the English questionnaire) skilled in English and Persian languages were asked to translate the Persian version of questionnaire into English independently. The English questionnaires translated by the research team and translators were reviewed and analyzed in one session, and the two English questionnaires were integrated to obtain a single temporary English version agreed upon by the research team and translators. This version of questionnaire along with the ambiguities and disagreements was sent to the developers of the original version for clarification and further explanation. This version was approved after applying their opinions (Fig. 1). To perform cognitive debriefing process, a pre-test to identify cognitive problems was inserted in the questionnaire. It was provided to 20 respiratory patients to resolve possible ambiguities to complicate questions, incorrect sentences, unnecessary questions, embarrassment or exhaustion caused to respondents and etc. and solutions for better comprehension of the questions were provided. Also, ten people of community were interviewed about their perception of the concept of questionnaire items. The results of these interviews were discussed in an expert committee consisting of a research team, eight lung diseases and health promotion specialists, one professional health specialist and one epidemiologist, and two English translators, and the necessary reforms were applied on the items. Finally, the final versions were prepared for the stage of reviewing psychometric properties. Table 2 presents translations of the items.

### Methods used to investigate validity and reliability

#### Face and content validity of the questionnaire

A group of 20 respiratory patients hospitalized and 10 professors (health promotion, professional health and epidemiology) were invited to evaluate the face validity and content validity of the questionnaire. Participants were given sufficient explanation about the aim of study and they expressed their consent to participate in the study. The questionnaires were given to the participants anonymously and voluntarily and they were asked to evaluate the items of the questionnaire in terms of comprehensibility, wording, interpretations, cultural issues and clarity of the questions. After applying the minor changes, quantitative content validity including content validity index (CVI) were assessed.

To evaluate CVI and CVR, 10 experts in the area of research (university professors) were interviewed. To review the CVI, experts were asked to examine the appropriateness of the area for each item. According to the guidelines, a CVI greater than 0.79 is appropriate, a CVI between 0.7 and 0.79 requires review, and a CVI less than 0.7 is unacceptable and the item should be deleted [19]. The CVI index was calculated using the following formula:

$${\text{CVI}} = \frac{{\text{The number of experts giving scores 3 or 4 to the item}}}{{\text{Total number of experts}}}$$

#### Discriminative validity

To assess discriminative validity, MRADL score of people with low work ability was compared to with the score of people with high ability using Mann–Whitney–Wilcoxon Test. The Work Ability Index (WAI) was used to assess work ability. The index was developed by the Finland Occupational Health Organization to assess and prevent early retirement and work-related disabilities. The validity and reliability of the Persian version of WAI were reviewed and confirmed by Makrami et al. [20]. WAI examines the ability to work in seven dimensions and the final score is the sum of the seven scores and is in the range of 7–49. A WAI score below 36 is classified as inappropriate or inadequate, and a WAI ≥ 37 is classified as appropriate [21]. It is assumed that people with poor performance scores have a lower mean MRADL score than people with appropriate work ability scores [2].

#### Convergent validity

Convergent validity was assessed by evaluating the relationship between MRADL score and emotional fatigue scores by calculating the Spearman's correlation coefficient. The fatigue score is expected to be highly correlated with MRADL score. The Fatigue Assessment Scale (FAS) was used to assess fatigue. This 10-item scale is dimensionless and has been developed from a combination of items from four fatigue questionnaires [22]. This scale assesses total fatigue. The psychometric properties of this instrument have been confirmed by Makrami et al. [23].

#### Reliability

Internal consistency of MRADL questionnaire was assessed using Cronbach's alpha index. Cronbach's alpha 0.9 or higher is considered very good, Cronbach's alpha 0.8–0.9 is considered good, Cronbach's alpha 0.7–0.8 is considered acceptable, Cronbach's alpha 0.6–0.7 debatable, Cronbach's alpha 0.5–0.6 is considered poor and Cronbach's alpha less than 0.5 is considered unacceptable [24].

## Results

### Demographic results of the study population

The mean age of participants was 48.8 ± 20.1 years. 176 (71.5%) were male, 166 (67.5%) were married and 116 (47.1%) had under diploma level of education (Table 1). The results showed that mean rank of the MRADL for males was 124.2 and 121.7 in females, but the difference was not statistically significant (*p* = 0.8). Concerning the relationship between the mean score of MRADL and marital status, the results showed that mean rank was 120.9 for married people and 128.7 for single people, which the observed differences were not statistically significant (*p* = 0.4).

**Table 1:** Frequency and percentage of demographic variables

Variable	Frequency (percentage)
Gender	
Male	176 (71.6)
Female	70 (28.6)
Marital status	
Single	80 (32.6)
Married	166 (67.5)
Living place	
Urban	205 (83.3)
Rural	41 (16.7)
Level of education	
Under diploma	116 (47.2)
Diploma	42 (17.1)
Bachelor	79 (31.8)
Upper and masters	9 (3.7)
Job status	
Physical	148 (60.2)
Psychological	37 (15.0)
Physical-psychological	61 (24.8)
BMI	
Underweight (less than 18.5)	20 (8.1)
Normal (18.5–25)	133 (54.1)
Overweight (25–30)	81 (32.9)
Obese (upper than 30)	12 (4.9)

### Cross-cultural adaptation

MRADL questionnaire items were modified according to intercultural and grammatical adaptations when translating into Persian.

During the step of translating MRADL into Persian, questions were asked and recommendations for changes were provided. The changes were applied based on the socio-cultural context and the changes were made with the approval of author of MRADL. Table 2 presents the Persian translation of the items, after which the back-translation was done. The items that needed to be changed and localized based on the existing socio-cultural context in Iranian society are stated below. In the original version of the questionnaire, there was “cross roads?” that changed to the item of do you cross the street since in Persian, the road can refer to intercity roads, while the street means the routes inside the city, which is more tangible and understandable, so in the final version, this change was applied

**Table 2:** The Manchester respiratory activities of daily living questionnaire items to which changes were made after their translation and cross-cultural adaptation into Persian language

Original question	Forward translation	Backward translation
Do you walk outside?		Do you walk outside?
You go up the stairs		You go up the stairs
You get in or out of the car		You get in or out of the car
You walk on uneven ground		You walk on uneven ground
Do you cross the street?		Do you cross the street?
You travel by public transport		You travel by public transport
Do you bend over to stand?		Do you bend while standing (for better breathing)?
Do you take things off the shelf above your shoulder height?		Do you take things off the shelf above your shoulder height?
You carry hot drinks from room to another room		You carry hot drinks from room to another room
Do you do washing work?		Do you do washing work?
Do you prepare hot food yourself?		Do you prepare hot food yourself?
You do the general housework		You do the general work of the house
Do you wash your small clothes? (Underwear, socks, etc.)		Do you wash your underwear yourself? (Underwear, socks, etc.)
Do you buy yourself?		Do you buy yourself?
Do you wash all your clothes?		Do you wash all your own clothes?
You do your personal hygiene (brushing, etc.)		You do your personal hygiene (brushing, etc.)
You take a bath yourself		You bathe alone
Do you go out for social interactions?		Do you go out for social interactions?
Do you do your own gardening?		Do you do your own gardening?
Do you have to eat slower than you like?		Do you have to eat slower than you like?
Does your breathing wake you up during the night?		Does your breathing wake you up during the night?

Also, in the original questionnaire, the item of “Wash small items of clothing” changed to “Do you wash your small clothes” in back-translation (Underwear, socks, etc.), which not only conveys the meaning of the main question, but also expresses the minor clothes in parentheses to guide the respondent to items such as socks, etc. Also, the item of “make yourself a hot snack” changed to “do you prepare hot food yourself”. Also, the item of “wash and dry yourself” changed to “you do your personal hygiene (brushing, etc.) (like brushing). The item of “have a bath” also changed to “you take a bath yourself” and the item of “go out socially” changed to “do you go out for social interactions” to better express the concept and adapt to the socio-cultural context. Also the item of “manage your own garden” changed to “do you do your own gardening” since having a garden is not common in many Iranian homes.

#### Validity

The total mean scores of CVI values ​​of the scale were 0.82, indicating the excellent content of the scale from the experts’point of view.

The mean MRADL score was significantly lower in people with poor working ability (mean = 8.86, SD = 5.07) compared to people with good working ability (mean = 15.81, SD = 3.13) (*p* < 0.001). These results suggest appropriate discriminative validity of MRADL. The results of Spearman's rho analysis showed that there was a very high positive correlation between MRADL score and WAI score (r = 0.73, *p* < 0.001). Also, this statistical test showed a high negative correlation between MRADL score and fatigue score (r = 0.58, *p* < 0.001). These results indicated appropriate convergent validity of MRADL.

#### Reliability

Results revealed that MRADL had a very good internal consistency, as its Cronbach's alpha coefficient was obtained at 0.90. Also, the correlation of each item of scale with the total score of scale was calculated and it was found that the correlation of all items with the total score of scale was significant at the statistical level *p* < 0.001. In other words, all items of the questionnaire had the needed consistency. Table 3 presents the results of corrected Item-total correlation, and Cronbach’s alpha if item deleted in all MRADL items. This table shows that all items are necessary in the questionnaire, and by deleting any of them, Cronbach's alpha of the questionnaire will not change significantly.

**Table 3:** Average results, corrected item-total correlation and Cronbach's alpha if item deleted for all MRADL items

	Scale mean if item deleted	Scale variance if item deleted	Corrected item-total correlation	Cronbach's alpha if item deleted
Do you walk outside?	9.6382	30.860	0.609	0.888
You go up the stairs	9.5772	30.498	0.668	0.886
You get in or out of the car	9.4146	30.938	0.606	0.888
You walk on uneven ground	9.5447	31.188	0.535	0.890
Do you cross the street?	9.5407	30.862	0.597	0.888
You travel by public transport	9.6626	32.657	0.277	0.897
Do you bend over to stand?	9.6098	33.374	0.142	0.901
Do you take things off the shelf above your shoulder height?	9.4350	30.728	0.640	0.887
You carry hot drinks from room to another room	9.5081	30.790	0.612	0.888
Do you do washing work?	9.6585	31.965	0.405	0.894
Do you prepare hot food yourself?	9.6260	31.566	0.472	0.892
You do the general housework	9.6138	31.022	0.573	0.889
Do you wash your small clothes? (Underwear, socks, etc.)	9.7033	31.279	0.551	0.890
Do you buy yourself?	9.5691	31.299	0.515	0.891
Do you wash all your clothes?	9.7114	32.263	0.363	0.895
You do your personal hygiene (brushing, etc.)	9.3862	30.491	0.708	0.886
You take a bath yourself	9.3862	30.491	0.708	0.886
Do you go out for social interactions?	9.5732	31.397	0.497	0.891
Do you do your own gardening?	9.6301	32.887	0.230	0.898
Do you have to eat slower than you like?	9.4837	30.790	0.616	0.888
Does your breathing wake you up during the night?	9.6220	31.983	0.394	0.894

## Discussion

This is a cross-cultural study which was performed to translate and cross-cultural adaptation of the MRADL questionnaire in Persian and to examine its psychometric properties. The process of cultural localization of the questionnaire was performed based on a standard and valid process. Psychometric properties of the instrument were confirmed based on face and content validity assessments, convergent validity, discriminative validity and internal consistency. The aim of assessing face validity and content validity was to answer the question of whether the face and content of the instrument were properly designed to evaluate the aim of the study. In this process, from the point of view of respiratory patients, health education specialists, lung diseases, professional health and epidemiology were used and their corrective opinions were applied to improve the quality and facilitate sentence comprehension. Quantitative content validity based on CVI indicated good content validity of the questionnaire items [22]. One of the problems in the process of cultural localization of questionnaire in Persian was that some questions were not appropriate to socio-cultural context of Iranian society. For example, the existence of gardens in urban homes is not common in Iran, and this question should be changed based on cultural activities of the Iranian context. These changes may be applied to create cultural consistency in the translation of different questionnaires, as has been reported in Brazil [25].

One of the changes made in the translated version due to cultural considerations, we can refer to the word “roads” in the item of cross roads, sine in Persian, road can give the meaning of intercity roads, while street means inner city routes, which is more tangible and comprehensible, and in the final translation, it changed to do you cross the street? Also, the item of “make yourself a hot snack” changed to “do you prepare hot food yourself” that indicates fast food since snacks are not cooked in Iranian culture and these people are not familiar with the word snack and it might make people give false to answer the question. In the evaluation of target group, they comprehended these items well and evaluated them as easy and comprehensible items; also to better comprehend, the item of “go out socially” changed to “do you go out for social interactions”. The item of “manage you own garden” changed to “do you do your own gardening” since having a garden is not common in many Iranian homes. The reliability of the questionnaire was assessed using Cronbach's alpha coefficient to evaluate internal consistency of the questionnaire. Cronbach’s alpha coefficient of the questionnaire was obtained at 0.9, which was very close to Cronbach’s alpha coefficient of 0.91 in the original questionnaire [12], and other similar studies conducted in Brazil (0.92) [25]. To examine the internal correlation of scale accurately, item-total correlation of the questionnaire items was assessed.

These analyses showed that almost all items had a good correlation with the total MRADL score, which explains the appropriate internal consistency of the scale. The only item that was less correlated with others items was the item of “Do you bend while standing (for better breathing)?. This low correlation might be due to the study population in the present study; because majority of the participants were male (71.6%) with mean age 48.8 ± 20.1; that they were at working age and they have not yet lost their work ability. The results showed that there was a significant relationship between them; This correlation can be a good discriminative index of the work ability and the score of respiratory activity, so that people who have more daily respiratory activity have a higher work ability.

Also, the results showed that there was a significant inverse relationship between fatigue and respiratory activity score. In a similar studies there were a significant inverse relationship between the work ability and fatigue [26–28]. In this regard, it can be said that those who have a higher ability to do work, have a better respiratory status in daily activities because their respiratory problems can be one of the important indicators in reducing the ability to work in people; People who report higher fatigue are more likely to have more respiratory problems, and it seems logical that there is an inverse relationship between fatigue from work and the ability to perform daily breathing activities.

## Conclusion

Results of the present study revealed that the translated version of the MRADL questionnaire has good validity and reliability indices and discriminative power also so that it can be used in future studies as an efficient and valuable instrument. Translating the questionnaire into the target language and translating it into the original language and comparing them with each other showed that the process of translating the questionnaire in accordance with the cultural principles and the target language was well done. Also, given its shortness and conciseness, it can provide useful and accurate results. This instrument has a desirable comprehensiveness by including four important areas of life activities that can be easily used in hospital wards and especially for patients with severe respiratory problems.

## Data Availability

The authors declare that all the raw data related to this research has been sent in the main manuscript file, in other word a submission to the journal implies that materials described in the manuscript, including all relevant raw data, will be freely available to any researcher wishing to use them for non-commercial purposes, without breaching participant confidentiality.

## Abbreviations

MRADL: Manchester respiratory activities of daily living questionnaire
CVI: Content validity index
CVR: Content validity ratio
COPD: Chronic obstructive pulmonary disease

## References

[1] Organization WH, Initiative TF (2007). Protection from exposure to second-hand tobacco smoke: policy recommendations.

[2] Monjazebi F, Dalvandi A, Ebadi A, Khankeh H, Rahgozar M, Richter J (2014). Psychometric properties of instruments measuring activities of daily living in patients with copd: a systematic review. COPD.

[3] Costa JIM. Use of a smartphone for self-management of pulmonary diseases. 2015.

[4] Pinnock H, Kendall M, Murray SA (2011). Living and dying with severe chronic obstructive pulmonary disease: multi-perspective longitudinal qualitative study. BMJ.

[5] Bestall J, Paul E, Garrod R, Garnham R, Jones P, Wedzicha JJT (1999). Usefulness of the medical research council (MRC) dyspnoea scale as a measure of disability in patients with chronic obstructive pulmonary disease. Thorax.

[6] Restrick L, Paul E, Braid G, Cullinan P, Moore-Gillon J, Wedzicha JJT (1993). Assessment and follow up of patients prescribed long term oxygen treatment. Thorax.

[7] Dewan N, MacDermid JC, Packham TJCRP, Medicine R (2013). Role of a self-efficacy-based model of intervention: the LEARN approach in rehabilitation of distal radius fracture. Crit Rev Phys Rehabil Med.

[8] Bossenbroek L, de Greef MH, Wempe JB, Krijnen WP, Ten Hacken NH (2011). Daily physical activity in patients with chronic obstructive pulmonary disease: a systematic review. COPD J Chronic Obstr Pulm Dis.

[9] De Vriendt P, Gorus E, Cornelis E, Velghe A, Petrovic M, Mets T (2012). The process of decline in advanced activities of daily living: a qualitative explorative study in mild cognitive impairment. Int Psychogeriatr.

[10] Stull DE, Kline Leidy N, Jones PW, Ståhl E (2007). Measuring functional performance in patients with COPD: a discussion of patient-reported outcome measures. Curr Med Res Opin.

[11] Yohannes AM, Roomi J, Winn S, Connolly MJ (2000). The Manchester respiratory activities of daily living questionnaire: development, reliability, validity, and responsiveness to pulmonary rehabilitation. J Am Geriat Soc.

[12] Yohannes AM, Greenwood YA, Connolly MJ (2002). Reliability of the Manchester respiratory activities of daily living questionnaire as a postal questionnaire. Age Ageing.

[13] Junkes-Cunha M, Mayer AF, Reis C, Yohannes AM, Maurici R (2016). The Manchester respiratory activities of daily living questionnaire for use in COPD patients: translation into Portuguese and cross-cultural adaptation for use in Brazil. J Bras Pneumol.

[14] Silva GPFD. Impacto da reabilitação pulmonar no coping religioso e religiosidade de pacientes com doença pulmonar obstrutiva crônica. 2017.

[15] Mathias S, Fifer S, Patrick D (1994). Rapid translation of quality of life measures for international clinical trials: avoiding errors in the minimalist approach. Qual Life Res.

[16] Akbaritabar AA, Mokarami H, Nazifi M, Rahi A, Mirkamandar E, Hosseinpouri M (2013). Psychometric properties of Spector's job satisfaction survey in the Iranian population. Koomesh.

[17] Maasoumi R, Mokarami H, Nazifi M (2017). Psychometric properties of the Persian translation of the sexual quality of life–male questionnaire. Am J Mens Health.

[18] Hobfoll SE (2011). Conservation of resource caravans and engaged settings. J Occup Organ Psychol.

[19] Polit DF, Beck CT, Owen SV (2007). Is the CVI an acceptable indicator of content validity? Appraisal and recommendations. Res Nurs Health.

[20] Mokarami H, Mortazavi SB, Asgari A, Choobineh A, Stallones L (2017). Multiple dimensions of work-related risk factors and their relationship to work ability among industrial workers in Iran. Int J Occup Saf Ergonom.

[21] Mokarami H, Toderi S, Rahimi Pordanjani T, Taban EJ (2018). Role of psychosocial job stressors on sexual function of male nurses: The mediator role of work ability. Am J Men's Health.

[22] Michielsen HJ, De Vries J, Van Heck GL, Van de Vijver FJ, Sijtsma K (2004). Examination of the dimensionality of fatigue. Eur J Psychol Assess.

[23] Mokarami H (2020). Developing a model to explain turnover intention based on macroergonomics factors and role of fatigue and work ability index (WAI) as mediators.

[24] Jain S, Angural V (2017). Use of Cronbach's alpha in dental research. Med Res Chron.

[25] Junkes-Cunha M, Mayer AF, Reis C, Yohannes AM, Maurici R (2016). The Manchester respiratory activities of daily living questionnaire for use in COPD patients: translation into Portuguese and cross-cultural adaptation for use in Brazil. J Bras Pneumol.

[26] da Silva FJ, Felli VEA, Martinez MC, Mininel VA, Ratier APP (2016). Association between work ability and fatigue in Brazilian nursing workers. Work.

[27] Wolvers M, Leensen M, Groeneveld I, Frings-Dresen M, De Boer A (2019). Longitudinal associations between fatigue and perceived work ability in cancer survivors. J Occup Rehabil.

[28] van Muijen P, Duijts S, Bonefaas-Groenewoud K, van der Beek A, Anema J (2017). Predictors of fatigue and work ability in cancer survivors. Occup Med.

